# Hospital Meetings, &c.

**Published:** 1900-03-24

**Authors:** 


### HOSPITAL MEETINGS, &C.

### LONDON HOSPITAL.

Mr. Sydney Holland, the chairman, presided at the quarterly court of governors of the London Hospital on the 7th inst., and read the report for the past year: In 1899 13,234 in-patients and 189,638 out-patients, making a total of 202,872 were treated at the hospital. These figures show an increase of 1,612 in-patients and 10,800 out-patients over the numbers treated in 1898. The daily average in the wards of beds occupied was 659, as against 656 for the preceding year. Six thousand nine hundred and sixty-six operations under anæsthetics were performed during the year, of which 2,508 were major operations. The audit of the accounts is not yet complete, but the committee are able to state that the total cash income of the hospital for 1899 was £93,000, and the total expenditure £94,000, showing an excess of expenditure over income of £1,000. We have again, he said, received £5,000 from the Prince of Wales's Hospital Fund, which is conditional upon an immediate and very necessary expenditure of £100,000 out of capital. The Hospital Sunday Fund made a grant of £5,000, and the Hospital Saturday Fund of £883. The Private Nursing Institution has been a great success, and the addition of 60 nurses has been amply justified. At the first meeting of the House Committee, elected at the court held on December 6th, Mr. Sydney Holland was again unanimously chosen chairman for the ensuing year. The committee had to report that a tender for the alteration of the front block of the hospital for £58,000, by Messrs. Perry and Co., Tredegar Works, Bow, has been accepted, and the work is in progress. This includes £13,000 for new operating theatre, a gift of an anonymous donor. The new pathological department, which is being built on the site of the old isolation wards, is well in hand, and is to be dedicated to the memory of Sir Andrew Clarke. A new operating theatre for obstetric cases has been built and is now in use. The temporary theatres which have been constructed for use during the rebuilding of the old one are almost ready. The temporary building for the use of out-patients was opened on Friday last, and has proved quite satisfactory. Everything now is ready for the building of the new out-patient department on the west side of Turner Street. At one end of the temporary wards which were built in 1899 in the garden a room has been fitted up for the treatment of lupus on the new system discovered by Professor Finsen, of Copenhagen. The Princess of Wales, who has always shown so great an interest in the hospital, has graciously presented a complete set of apparatus needed for this method. The thanks of the governors are also due to Dr. Stephen Mackenzie and Dr. J. H. Sequeira, who paid a visit to Copenhagen in order to inspect Professor Finsen's institute. The Princess of Wales paid a private visit to the hospital, spending three hours in the wards. Her Royal Highness expressed satisfaction with the arrangements made for the comfort of the patients. A freezing machine, which will enable us to manufacture our own ice at a very much less cost than we are able to buy it, and which will also prove valuable for cold storage and other hospital purposes, will be erected. The committee have also decided to advance to the Medical College a sum of money for the purchase of a sports ground and the erection of a pavilion. The Clubs Union have undertaken to pay 4 per cent. interest on the total sum required for the purchase of the ground, its laying out for cricket and football, and the building of a pavilion, to a limit of £5,000. The usual Christmas festivities were held on December 23rd. Mr. Edgar Speyer generously supplied presents for every patient in the hospital, numbering some seven hundred. The committee regret to report the death of Dr. Charlewood Turner, consulting physician of the hospital. To fill the vacancy thus made on the staff Dr.

Percy Kidd was appointed full physician on December 18th. The committee have every confidence in recommending Dr. Robert Hutchison, M.D., for election to the vacancy thus caused. The resignation of Dr. Cunningham, dental surgeon to the hospital, and the appointment of Mr. F. M. Farmer in his place have also to be reported. Mr. Hanbury, chairman of the Drug Committee since 1880, has resigned, and the committee propose to reconstruct the Drug Committee on a wider basis, thereby increasing its usefulness. The committee have to report the munificent gift of Mr. James Hora, a vice-president, of £6,000. The gift is conditional upon naming one of the new wards "Marie Celeste," in memory of Mr. Hora's late wife, and the committee have also called the temporary ward by the same name.

Among the donations received were three of £1,000, one of which, anonymous, was for telephones.

In reply to a question respecting the condition under which the grant from the Prince of Wales's Fund was made, the Chairman said they contemplated altogether a capital outlay of £250,000. The front block would cost £60,000; the out-patients' department, £60,000; isolation block, £30,000; postmortem and pathological department, £15,000; laundry, £15,000; internal alterations, £20,000; Jewish wards, £12,000; children's wards, £7,000; and so on. There was none of it for luxuries, but expenditure they must incur if they were to continue to go forward. They ought to be the leading hospital, and they intended to be.

The report was then adopted, and after a promise from the Chairman that the question of the signing of certificates for School Board children should receive proper attention, the meeting separated.

### HOSPITAL FOR DISEASES OF THE SKIN.

Sir James Dick presided at the annual meeting of the Hospital for Diseases of the Skin, Stamford Street, on Monday, 12th inst. The report of the Committee of Management stated that during the past year 5,397 new patients had received the benefits of the institution as compared with 4,392 in 1898, the total attendances numbering 13,551. As the reputation of the hospital had increased, the demands upon its accommodation had become more pressing, and reconstruction was considered imperative. With that view the committee had secured the renewal of the lease of the premises (though at an increased rental) and appealed for support to enable the improvements to be carried out. Meanwhile they had temporarily suspended the reception of in-patients. A laboratory had been fitted up, and thanks were tendered to Dr. Abraham and Dr. Marshall for the presentation of modern appliances for use in it. It was hoped that the field of research thus commenced would tend to the further usefulness of the hospital and the aid of science. Dr. Abraham, for many years assistant-surgeon, had been appointed surgeon, and Dr. Marshall, assistant-surgeon; while Dr. Payne, who had been compelled to sever his connection with the work of the hospital, had consented to become consulting physician. The financial statement showed the ordinary income of £1,023, and extraordinary income £1,111. The ordinary expenditure was £975, and the extraordinary expenditure (investment) £1,400. Regret was expressed at the loss by death of several old subscribers, among them the Duke of Beaufort, for many years a munificent supporter of the charity; on the other hand the hospital was last year included among the awards of the Hospital Sunday Fund. The amount contributed by out-patients towards the cost of their relief was £599, an increase of £125. The committee recorded their appreciation of the services of Mr. Richardson, the secretary, the improvement in management being abundantly evident in every department of the hospital. The report was adopted without discussion. The committee was re-elected with the addition of Dr. Marshall and Mr. Cutler, the honorary architect, and the proceedings terminated.

### THE NEW HOSPITAL FOR WOMEN.

The twenty-eighth annual meeting of the governors and subscribers of the New Hospital for Women, Euston Road, was held at the hospital on the 14th inst.

The Rev. Canon Scott Holland presided, and said that it gave him the greatest possible pleasure and refreshment to come and move the adoption of the report. The most surprising thing about hospitals was the sense of rest and refreshment imparted to those who came in contact with them. People outside were paralysed when confronted with disease, distress, and the burden of life; but when they crept, in fear and trembling, to the doors of the "homes of sorrow," they found them, contrary to their expectation, to be the brightest of places. The keynote, the spirit that predominated in all well-managed hospitals, was cheerfulness. No profession was so, might he say, "unwrinkled," as the medical profession. All others—the clergy, lawyers—bore on their faces the signs of toil and worry, but there seemed no sense of blight or burden in the work of a doctor. Then how bright and cheerful nurses always were! He saw, he observed, many of them at St. Paul's and in the streets, and it was always a pleasure to note their happy, purposeful mien, so different from the aimless indifference of the pleasure-seeking crowd. The workers in our hospitals, doctors and nurses, were indeed a great and happy race. Why was it, he asked, that when one gathered disease and sorrow home, housed and provided for it, that those who cared for it were brimming over with joy and gladness? Might not the reason be that so long as it is outside it thwarts and benumbs us, the very sight of it causing us to quiver like a horse in terror, our nerves all distraught, whilst in the hospital we go up to the thing, learn to know it, and with knowledge the horror vanished. Might it not be that facing the worst the worst became the best, and so a cheerful optimism pervaded the place. Whilst nothing was so irritating as a silly unreasoning optimism, nothing was so inspiriting as a logical optimism founded on fact. In a hospital people acted intelligently and beneficently, untroubled by the metaphysical problems that beset and hampered the workers outside. Doctors and nurses ask no questions, they are there to do the best for the patient, sure that health was to be won and that nature was on their side. The abnormal was the wrong—the accident—and they strove to rectify the evil. Therefore their optimism was justified, and the world responded to it, as it has ever responded to a creed proved by practical experiment. The faith and work in a hospital embodied the spirit of Christ. It appealed most strongly to women, for the first impulse of a woman confronted with disaster was to act promptly, with a clinging faith that right action would bring about good results. This hospital was an institution for women, founded and managed by women. It had met with a generous response on every side, though more especially from the poor, and he appealed to all who could afford it to support it with might and main. The great advantage in giving to a hospital was the certainty that every penny was wisely applied. Nothing clouded gaiety of heart so much as debt, and the authorities of this hospital had particularly sensitive consciences in this respect. So he asked those present to keep the faces of the lady doctors "unwrinkled," and to enable them and the nurses to go about their work without the carking burden of debt.

Mr. T. J. Dryhurst seconded the motion. He compared the number of patients, the receipts and expenditure for the last two years. The number of in-patients last year were 594; of out-patients, 7,377; whilst there were 598 new cases in the ophthalmic department. The maintenance fund amounted to £5,119 2s. 8d., and the expenditure came to

£5,319. The full accounts show a deficiency in the maintenance account of £120.

Sir Thomas Smith, Bart., F.R.C.S., moved that "Mrs. Sheppard, the Rev. H. L. Paget, and Mr. Henderson be re-elected to the committee, and Miss Freeman elected." He remarked that as he was now enjoying greater leisure he had lately been enabled to witness several operations in the hospital, and he had seen many very serious and dangerous operations performed with dexterity, celerity, and safety, with an accurate observance of the many details of antiseptic surgery. Many years ago he had declined to assist a lady surgeon at an operation because she did not intend to devote her life to that particular branch of surgery. He now was happy to do penance for his obstinacy, and to declare that he felt certain that for women women surgeons were the best; that nature had especially adapted them for the work by bestowing on them peculiar gifts and qualities. They had small hands, deftness, and were especially dexterous with the use of the needle and thread—skill which was no small advantage now that surgery was becoming more constructive. The criterion of all operations was in the results, and he had no fear of contradiction when he said that they were as good—and in some cases better—than those obtained in any other hospital in Great Britain.

The resolution was seconded by Mr. James Berry, F.R.C.S., who said a few words in praise of the medical side of the work carried on in the wards.

Mrs. Westlake proposed the election of the Hon. Maude Stanley as vice-president, and Mrs. Scharlieb, in seconding it, bore a warm testimony to her faithful services to the hospital. Mrs. Scharlieb took the opportunity of heartily eulogising the willing and competent work of the nursing superintendent and her staff. "We may as well," she said, "expect an army to get on without transport or commissariat as to expect surgeons to operate successfully without a willing and competent nursing staff."

Mrs. Garrett Anderson moved "that the rules relating to the election of the Managing Committee be rescinded, and that in future the election be by voting papers at the governor's election." This remodelling of the procedure would, it is hoped, be useful in introducing fresh blood into the counsels of the authorities.

Miss Cock seconded the resolution.

Mrs. Trenon was re-elected hon. auditor.

### WEST HAM HOSPITAL.

The annual general meeting of the governors and subscribers of the West Ham Hospital was held on Wednesday evening, 14th inst., at the Dispensary, West Ham Lane, E., Mr. George Hay, J.P., chairman of the Board of Management, presiding. The tenth annual report, which was read by the Secretary (Mr. Leonard D. Rea) stated that during 1899 636 in-patients were treated (523 being surgical and 113 medical cases), as compared with 744 in 1898. The average cost per week of each in-patient was £1 12s. 4d., against £1 14s. 3d. in 1898; whilst the average number of patients daily in hospital was 42, against 39 in 1898. In the out-patients' department 10,904 had been admitted by letter of recommendation, 9,299 casualties and cases of emergency were attended to, and also 1,833 dental cases, the total attendances of out-patients numbering 70,187. The list of the year's contributions included: Prince of Wales's Fund, £300 (annual grant) and £250 (special donation towards necessary repairs, &c.); Sunday Fund, £399; Saturday Fund, £130; School Children's Hospital Fund, £350. The hospital had been and continued to be in a financial condition giving rise to grave anxiety, and the committee had been obliged to seriously consider the necessity for closing one or two wards, and reducing the number of the staff. An appeal had been issued by Mr. Hay (the chairman) and was still open, which at the close of the year had produced a total of £684. A representative of the committee had been appointed to personally solicit and collect subscriptions, and this had resulted in a considerable number of new annual subscribers. Five beds had been placed at the disposal of the War Office for wounded soldiers returning from the war, and the offer had been accepted. The Treasurer (Mr. Duckett) then detailed the balance-sheet for the year. It showed that the total ordinary income had been £4,998, and the total extraordinary income only £19, making a total of £5,017. The total ordinary expenditure amounted to £4,715, and the extraordinary expenditure to £14, giving a total of £4,729. There was a deficit brought forward from 1898 of £263, and a deficiency on the year in addition of £276. The committee regretted that the £250 special donation from the Prince of Wales's Fund and £50 from the West Ham Corporation had had to be expended in meeting current expenses. This £300 will shortly have to be provided for payment of work now in hand, and for which the money was specifically given. The treasurer mentioned that the hospital had recently received a donation of £500, with which he had applied for an allotment of the new National Government (Khaki) Loan.

The Mayor of West Ham (Alderman Bethell) moved the adoption of the report and accounts, and in doing so suggested that ground rents in the borough would prove a better investment than the Khaki Loan. He further said that the hospital should have a convalescent home of its own. He put the suggestion forward for the consideration of the committee, and promised that if it was adopted he would undertake to raise subscriptions for that purpose to the extent of £5,000, and further undertook to see that a decent building was erected at the seaside if the matter was entertained. He suggested the purchase of forty-five acres of land at Southend for the purpose. The suggestion was warmly received.

Mr. Sharp seconded the motion for the adoption of the report, and it was agreed to.

### ROYAL HOSPITAL FOR DISEASES OF THE CHEST.

Alderman and Sheriff Sir William Treloar presided, in the absence of the Lord Mayor, at the eighty-sixth annual Court of Governors of the Royal Hospital for Diseases of the Chest, at the hospital, City Road, on the 15th inst. The report of the council for 1899 was presented and adopted. It stated that the in-patients numbered 798, as against 739 in 1898, while the out-patients' attendances were 26,542, as compared with 26,525. The ordinary income for the year was £6,730, and the extraordinary (legacies) £1,633, together £8,363. The ordinary expenditure was £7,339, extraordinary (building improvements) £151, together £7,491. Owing to the serious falling-off in the income during the early months of the year—the receipts from donations to the end of May being £741 only, against £4,459 for the corresponding period of last year—it was decided to make a special appeal for £2,000 to prevent the closing of one of the large wards. Miss Thompson, who resides in the neighbourhood, promised to give £500 if the other £1,500 had been subscribed by the end of June. In spite of every effort the amount was still £170 short at that date, but on learning this Miss Thompson handed the treasurer a cheque for £670, thus completing the £2,000. For the year the donations show a falling off of £2,006, but the annual subscriptions increased from £2,269 to £2,425, and legacies from £545 to £1,633. The expenditure showed a decrease of £990. Mr. Richards, M.P., in the re-election of the council, said he wished some men of wealth and position would come forward and identify themselves with so good a cause. He could point to half-a-dozen millionaires who made their fortunes in the City Road and the neighbourhood, but they never saw them, and in the name of charity could not get at them at all. Dr. Gilbert Smith, in seconding the resolution, said that in the din of war the needs of such an institution as theirs was apt to be overlooked. But the war in South Africa was passing; there they had a battlefield which was always with them; their foes were more insidious than the Boers, and though it was true the bacillus did not burst with the noise of a melanite shell, yet it was just as deadly. To combat that foe they must be provided with the sinews of war. Colonel Makins moved a very hearty vote of thanks to the honorary medical officers, who he said were the mainspring and the backbone of the institution. The governors gave money for its support, the council gave time and money, but the medical staff gave time and money and skill. The Chairman having promised to do what he could to induce the City Corporation to assist the institution, the meeting closed with the customary votes of thanks.

